# Supplementary material for: m6AnetAnalyzer: an R toolkit for post-processing of m6A sites detected by m6Anet
Source: Bioinform Adv. 2026 Mar 26;6(1):vbag089. doi: 10.1093/bioadv/vbag089 (PMC13069878; doi:10.1093/bioadv/vbag089)
Supplement: vbag089_Supplementary_Data [file vbag089_supplementary_data.pdf]

A

Condition	Total Sites	Total Transcripts	Total Genes	>.90 Sites	>.90 Transcripts	>.90 Genes
IGF2BP2 KD	257808	17627	4976	4228	2796	1067
Naive	526317	24438	6773	10605	5710	2047

B

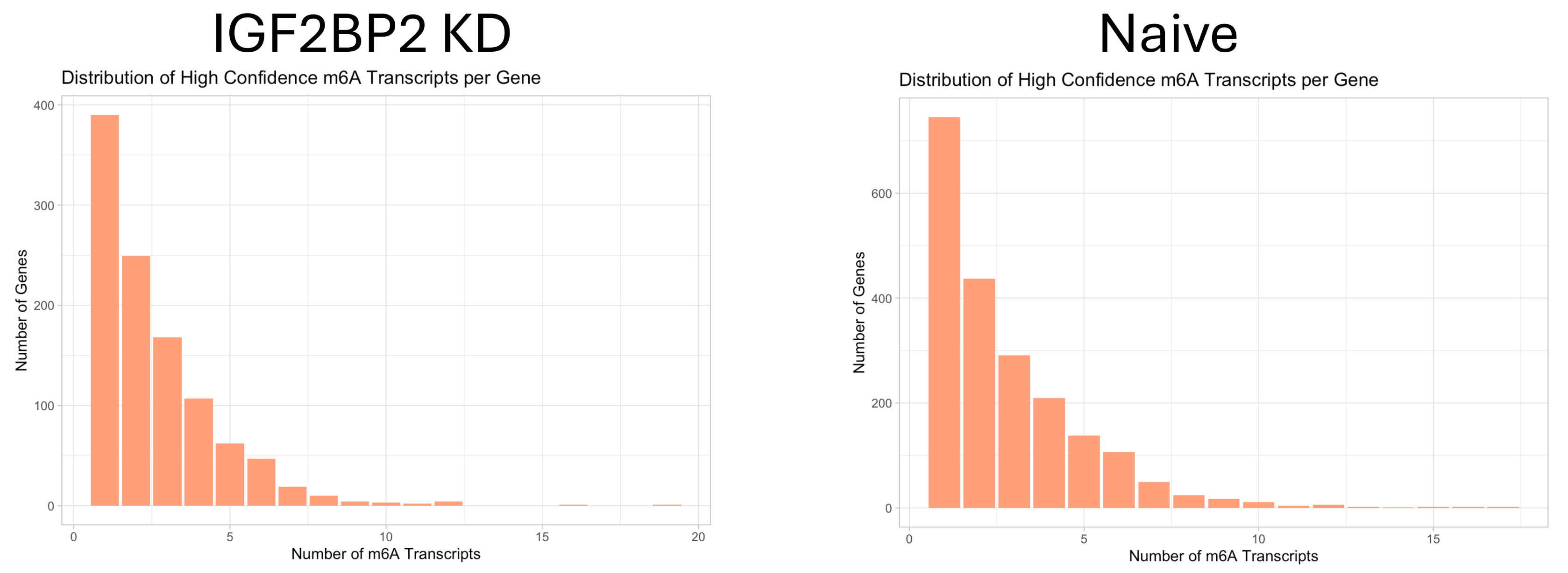

C

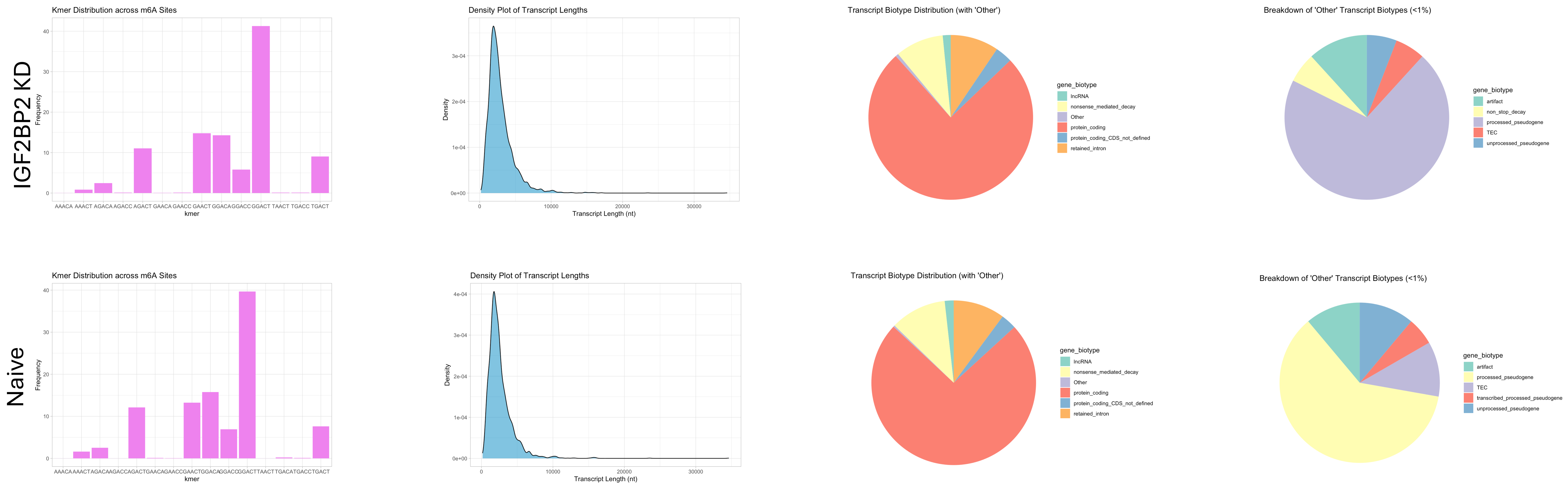

D

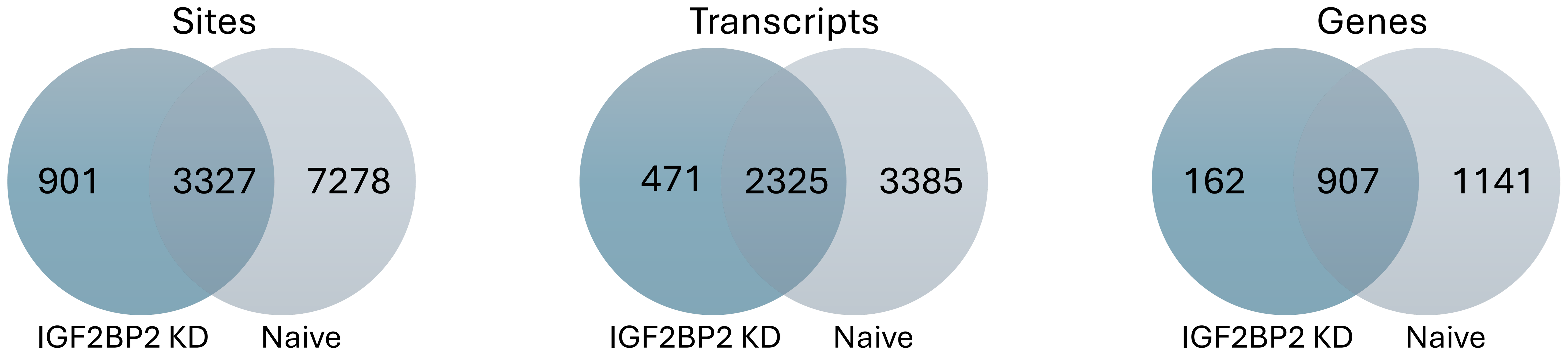

Supplementary Fig. 1. m6ASeqTools Output for IGF2BP2 KD and Naïve conditions. (a) Table of total and high-confidence (probability_modified > .9) m6A sites, transcripts and genes. (b) Distribution of the number of genes with associated counts of modified transcripts per gene. (c) Descriptive statistics of m6A modifications for IGF2BP2 (top) and Naïve (bottom), including kmer distribution, modified transcript length and biotype distribution. Pie chart on the left shows the frequency of modified transcript biotypes with "Other" category, which is then broken down in pie chart on the right. (d) Venn Diagrams showing the distribution of common and unique sites (left), transcripts (middle) and genes (right) between the two conditions.

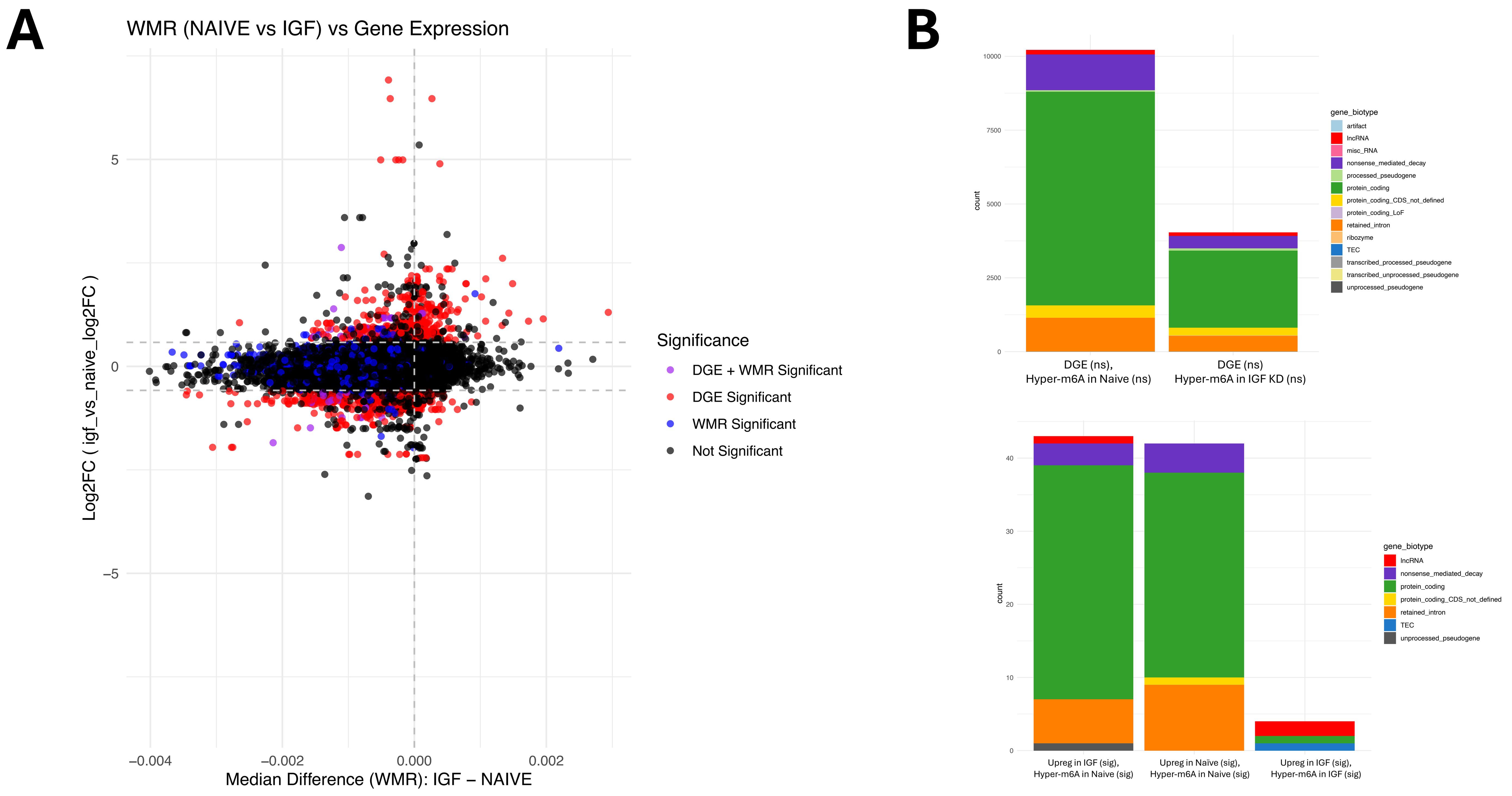

Supplementary Fig. 2. Differential outcomes for IGF2BP2 knockdown (KD) and naïve conditions. (a) Scatter plot showing the relationship between median weighted modification ratio differences and gene expression. Red: significant change in expression (|log2FC| > 0.58, $p < 0.05$). Blue: significant difference in weighted modification ratios (Wilcoxon rank-sum test, $p < 0.1$). Purple: significant for both gene expression and methylation. (b) Biotype distribution of clusters with differential methylation and/or expression.
